# Fascial nomenclature: Update on related consensus process

**DOI:** 10.1002/ca.23423

**Published:** 2019-06-27

**Authors:** Robert Schleip, Gil Hedley, Can A. Yucesoy

**Affiliations:** ^1^ Department of Neuroanesthesiology Neurosurgical Clinic, Ulm University Guenzburg Germany; ^2^ Department of Sports Medicine and Health Promotion Friedrich Schiller University Jena Jena Germany; ^3^ Fascia Research Group, Experimental Anesthesiology Ulm University Ulm Germany; ^4^ Integral Anatomy Productions LLC Melbourne Florida; ^5^ Biomedical Engineering Bogazici University Istanbul Turkey

**Keywords:** fascia, terminology, Delphi technique, nomenclature, connective tissue

## Abstract

The term fascia is increasingly used not only by anatomists but also by other professionals and authors in different health‐oriented fields. This goes along with an inconsistent usage of the term, in which many different tissues are included by different authors causing an increasing amount of confusion. The Fascia Research Society acted to address this issue by establishing a Fascia Nomenclature Committee (FNC) with the purpose of clarifying the terminology relating to fascia. This committee conducted an elaborate Delphi process to foster a structured consensus debate among different experts in the field. This process led to two distinct terminology recommendations from the FNC, defining the terms “a fascia” and “the fascial system.” This article reports on the process behind this proposed terminology as well as the implications for inclusion and exclusion of different tissue types to these definitions. Clin. Anat. 32:929–933, 2019. © 2019 The Authors. *Clinical Anatomy* published by Wiley Periodicals, Inc. on behalf of American Association of Clinical Anatomists.

## INTRODUCTION

Hardly any area of anatomical science is characterized by such divergent terminology as is the case in the field of fascia‐related connective tissues. For many experts in the field, only dense sheet‐like connective tissues are considered as “fascia,” and only if they express more than one dominant fiber direction. Consequently, for a connective tissue to be regarded as fascia, its fiber arrangement is often considered to be “irregular.” However, such inference may be incorrect, particularly if, for example, epimysial envelopes are considered in which tissues, two main fiber directions are present that cross each other in a regular manner at very specific angles (Benetazzo et al., 2011).

In contrast, other authors in this field also include very soft layers such as the areolar zones within the hypodermis or as is found in the envelopes around tiny vessels (Guimberteau and Armstrong, 2015). Some authors restrict the term “fascia” to muscular connective tissues (Landers, 2019). Visceral connective tissues—like the mediastinum, the pericardium or the mesentery root—are then excluded. In contrast, more osteopathic‐oriented textbooks put great emphasis on the visceral fasciae (Paoletti, 2006; Schwind, 2006). Similarly, there has been confusion about the question as to which of the three hierarchical muscular tissue containers–epimysium, perimysium, and endomysium–could be included as “fascia.” While most anatomists tend to agree to consider muscular septi and the perimysium to be fascial tissues, there is less consensus on the endomysium due to its microscopic size and/or a higher quantity of collagen types III and IV which are also associated with a softer tissue structure. The resultant confusion in language yields major difficulty in communication between different professionals in the field. Additionally, the lack of clarity in the terminology detracts from specifying, scientifically/clinically addressing and communication of functionally important aspects of fasciae. For example, muscular connective tissues have been shown to affect muscle function (Wilke et al., 2018) which indicates several clinical implications (Yucesoy and Huijing, 2007) and endomysium, as an integral part of this system plays a central role determining the muscle's contribution to joint movement (Huijing, 1999).

Several attempts have already been made by respected international institutions to respond to this challenging situation. The International Anatomical Nomenclature Committee (1983) confirmed the usage of previous nomenclature committees and used the term “fascia superficialis” for the entire loose layer of subcutaneous tissue lying superficial to the denser layer of “fascia profunda.” While most medical authors in English‐speaking countries followed that terminology, authors in other countries did not congruently adopt it. For example, many Italian authors excluded the panniculus adiposus situated within this tissue layer, and most French authors continued to exclude both the panniculus adiposus and the textus connectivus laxus beneath the stratum membranosum (Wendell‐Smith, 1997).

The subsequent international nomenclature, proposed by the Federative Committee on Anatomical Terminology (1998), therefore attempted to lead toward a more uniform international language (Wendell‐Smith, 1997). It defined fascia as “sheaths, sheets or other dissectible connective tissue aggregations.” This includes “investments of viscera and dissectible structures related to them.” This highly esteemed group of anatomical experts suggested that future authors should no longer use the term “fascia” for loose connective tissue layers and should instead apply the term “fascia” only to denser connective tissue aggregations. Accordingly, they recommended against the use of the old term “superficial fascia” as such (and to substitute “tela subcutanea” or “subcutaneous tissue”). Congruent with this decision, this most recent international *Terminologia Anatomica* even suggested excluding some of the most frequently used “fascia” names in anatomy from their proposed definition. For example, they recommended that the commonly used term “Camper's fascia” should be abandoned and be replaced by the term “panniculus adiposus abdominis” (FCAT, 1998).

This elegant attempt for the most part failed (Huijing and Langevin, 2009). Many English textbooks continued to use the terms “superficial fascia” or “Camper's fascia” (Platzer, 2008; Netter, 2011; Tank, 2012). This included the 39th edition of *Gray's Anatomy* (Standring, 2008), while the following 40th edition started to follow the fascia‐related recommendations of the *Terminologia Anatomica* (Standring, 2015). In contrast, the recommended terminologies in the publications around the Fascia Research Congress lineage (Findley and Schleip, 2007; Huijing et al., 2009; Chaitow et al., 2012; Wearing et al., 2015) do include tissues such as joint capsules, loose connective tissues, ligaments, and aponeuroses.

The critique of the latter group of authors has been well formulated regarding the proposed distinction (in the *Terminologia Anatomica* as well as *Gray's Anatomy*) between fasciae and aponeuroses (Schleip et al., 2012). While such differentiation is easily possible in areas such as the human lower back (Benjamin, 2009; Willard et al., 2012), it becomes very cumbersome in other parts of the body, which express various transitions between unidirectional and multidirectional textures, which is very often the case in the vicinity of major joints. In fact, as shown by the work of van der Wal (2009), tendons and aponeuroses often do not insert directly into the skeleton; instead, they tend to blend and connect with capsular and ligamentous tissues close to their attachments.

**Figure 1:**
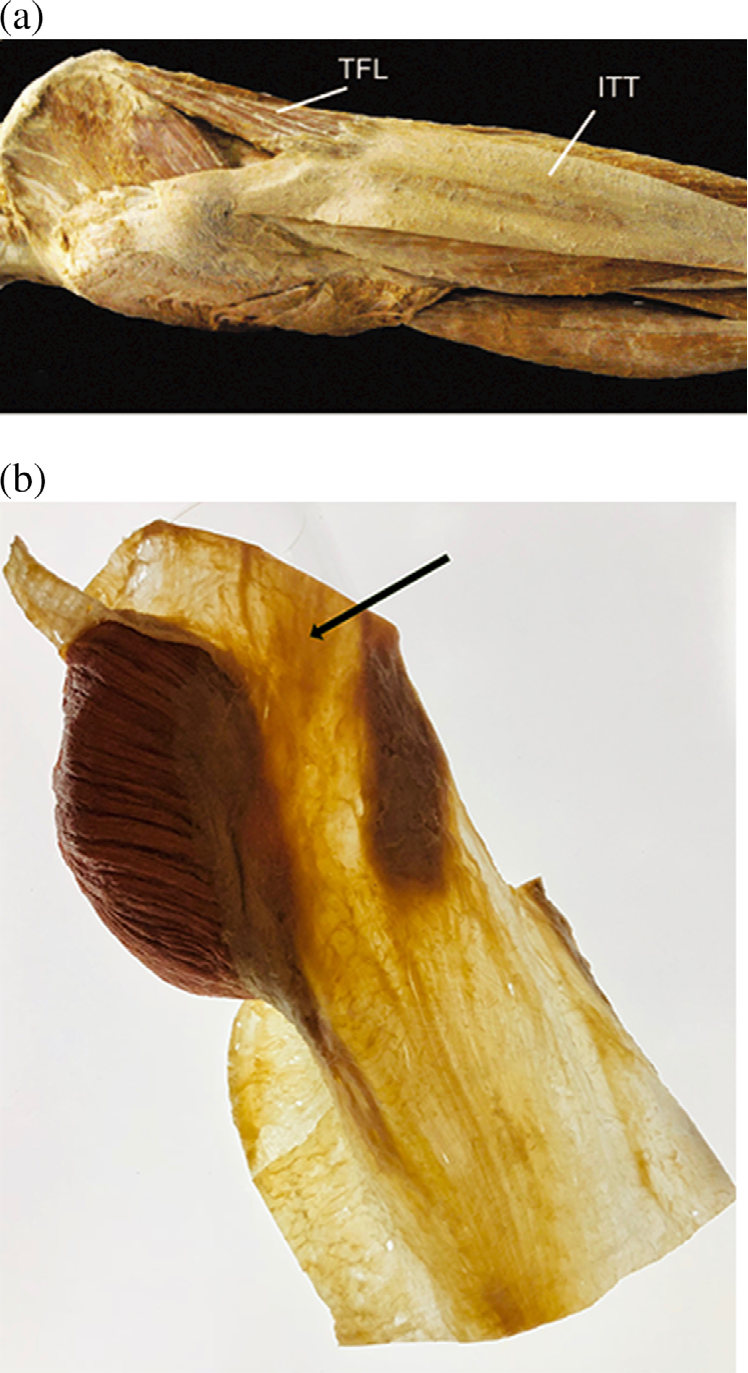
(**A**) Example of a fascia dissection based on medically “precise” terminology. This dissection image was used in an otherwise excellent treatise on the iliotibial tract (ITT). Following the proposal of Gray's Anatomy (Standring, 2008) to distinguish between aponeuroses and fasciae, the authors chose to describe this tissue as an aponeurosis and therefore excluded all tissue portions with a non‐aponeurotic character. Unfortunately, this included one of the sturdiest portions of this structure: the connection to the lateral iliac crest, posterior of the anterior superior iliac spine. Notice the common thickening of the iliac crest at the former attachment of this ligamentous portion (located at a straight force transmission line from the knee over the greater trochanter), reflecting the very strong pull of this “ligamentous portion” of the ITT on the pelvis. TFL, tensor fasciae latae. (**B**) Dissection of the same structure based on the functional term “the fascial system.” The strong densification of the “ligamentous portion” of the ITT on this preparation can be easily recognized, indicated by the arrowhead. In addition, note the continuous transitions on the ITT between regions with a unidirectional and others with a multidirectional fiber orientation. The specimen is one of the first samples of the Fascial Net Plastination Projection of the Fascia Research Society, in which a three‐dimensional plastinated demonstration of “the fascial net” of the human body is attempted. (A) Illustration taken with permission from Benjamin et al. (2008). (B) Illustration ©http://fasciaresearchsociety.org/plastination, with support from Gubener Plastinate GmbH. [Color figure can be viewed at http://wileyonlinelibrary.com]

Figure 1A illustrates a description of the iliotibial band in which the respective authors attempted to apply proper terminology (in their case with multiple references to *Gray's Anatomy*) and to use the term “aponeurosis”—as distinguished from other dense connective tissue bands and sheets–for dense connective tissue sheets which can be seen as direct extensions of skeletal muscle fibers (Benjamin et al., 2008). In congruence with this clear terminological distinction, the authors went ahead and excluded (and even excerpted) one of the sturdiest pieces in their otherwise exemplary analysis of the iliotibial band because it did not fit their nomenclature. However, as can be seen on a novel anatomical dissection of the same structure shown in Figure 1B, the tissue portion excerpted by the previous investigation constitutes one of the sturdiest elements of the upper leg and obviously plays a major role in the tensional force‐transmitting function of the iliotibial band. It seems likely that any subsequent analysis of the biomechanical function of the iliotibial tract will tend to be misleading if this important element is excluded. In fact, it seems that while using their scalpel in perfect adherence to the terminological distinctions of *Terminologia Anatomica* and *Gray's Anatomy*, the authors discarded one of the most important force‐transmitting elements from this structure.

Based on this and many similar points of critique on the existing situation and on the increasing confusion of terms (Stecco, 2014), the creation of a task force was suggested as a useful step toward building a consensus (Langevin, 2014). The proposed steps include the following:

1. “diverse points of view need to be heard. This means that the task force should include representatives from major stakeholders (i.e., individuals and groups who have already published in this area)
2. individuals who are not part of the task force need to have the opportunity to voice their opinions …
3. consensus needs to be reached within the task force
4. recommendations need to be clear and published in such a way that people who are new to the field can easily find them…” (Langevin, 2014).

The Fascia Research Society acted to address this issue by establishing a Fascia Nomenclature Committee (FNC) in mid‐2014. This article reports the activities and resultant terminological recommendation from this group.

## MATERIALS AND METHODS

This group quickly reached out to all authors known to them who had published on this topic in the English language in academic journals to invite them for active participation. In addition, the group decided to apply the Delphi method as a structured and transparent communication process for fostering a statement of consensus among a wide array of experts in a specific field (Adler and Ziglio, 1996; von der Gracht, 2012). The process included three written communication rounds, each of which consisting of a questionnaire being distributed to the experts, collecting and summarizing their responses, and communicating this back to the same group. The participants were always allowed to comment on the responses of others, as well as on the proposed summaries from the facilitators. A total of 21 experts participated in the three rounds of this process.

During the first two rounds, it became clear that, given the wide range and disparity of perspectives and linguistic traditions among the different professionals involved, it would not be possible, even with multiple additional rounds of communication, to reach a shared consensus about a single recommended usage of the term “fascia.” The ongoing process, therefore, aimed at a possible consensus regarding several different and alternate definitions instead.

The third round of the Delphi process was already structured as a preparation of a personal committee meeting, being held in association with the 4th Fascia Research Congress, Washington, 2015. Fifteen of the previously participating experts, as well as four nonvoting external guests, attended this meeting. As a major new step toward achieving a comprehensive and practical terminology, it was decided to establish two different fascia‐related definitions. One of those was proposed toward detailed and distinction‐oriented histological descriptions, whereas the other definition aims to emphasize the uniting character of the fascial net by recognizing the multijoint functional capacities of this body‐wide continuous network. A clear formulation for the former definition was already achieved at this face‐to‐face meeting. A special task force was created at the same meeting with the aim of elaborating on the formulation of the later definition with continuing input from the larger group of experts. This report covers the final consensus of the FNC regarding both of the proposed definitions.

## RESULTS

The FNC delivered two different terminologies based on different classifications. The first one—centered around the term “a fascia”—is recommended for communication of histological and topographical aspects on a mesoscopic and microscopic scale. In contrast, the second terminology—using the term “the fascial system”—is recommended for the description of functional properties on a macroscopic scale. Such functional properties include force transmission, sensory functions (proprioception, interoception, and nociception), fluid transmission, as well as the regulation of wound healing and fibrotic pathological processes (Tables 1 and 2).

**Table 1:** *Proposed histological/anatomical definition*, suggested by the FNC (Stecco and Schleip, 2016; Stecco et al., 2018)

*A fascia* is a sheath, a sheet, or any other dissectible aggregations of connective tissue that forms beneath the skin to attach, enclose, and separate muscles and other internal organs.

**Table 2:** *Proposed functional definition*, suggested by the FNC (Adstrum et al., 2017)

*The fascial system* consists of the three‐dimensional continuum of soft, collagen containing, loose and dense fibrous connective tissues that permeate the body. It incorporates elements such as adipose tissue, adventitiae and neurovascular sheaths, aponeuroses, deep and superficial fasciae, epineurium, joint capsules, ligaments, membranes, meninges, myofascial expansions, periostea, retinacula, septa, tendons, visceral fasciae, and all the intramuscular and intermuscular connective tissues including endomysium/perimysium/epimysium. The fascial system surrounds, interweaves between, and interpenetrates all organs, muscles, bones, and nerve fibers, endowing the body with a functional structure, and providing an environment that enables all body systems to operate in an integrated manner.

## DISCUSSION

While the recommended terms “a fascia” and “the fascial system” may not always fit well into the syntax of a given specific linguistic context, the more conventional terms “proper fascia” and “fascial tissues” may sometimes serve as useful replacements.

The definition “a fascia” is very closely oriented on the most recent fascia definition of the *Terminologia Anatomica*. Here, only planar tissues that can be dissected with a conventional scalpel are included. In contrast, tissues like the endomysium or tendons, which do not fulfill this criterion, are excluded.

The second term “the fascial system” acknowledges the increasingly popular concept of fascia as a body‐wide interconnected and prestretched fibrous network that is characterized—at least to some degree—by the expression of tensegrity properties (Findley, 2011). Here, all fibrous connective tissues are included, which can be seen as elements of a body‐wide tensional force‐transmission system also including ligaments, tendons, joint capsules, and intramuscular connective tissues. It could be argued that the term “the fascial system” may then be synonymous to the term “connective tissue.” However, the newly proposed term differentiates from the established medical terminology, in which, the term “connective tissue” clearly includes bones, cartilage, and even blood as former mesenchymal tissues.

**Figure 2:**
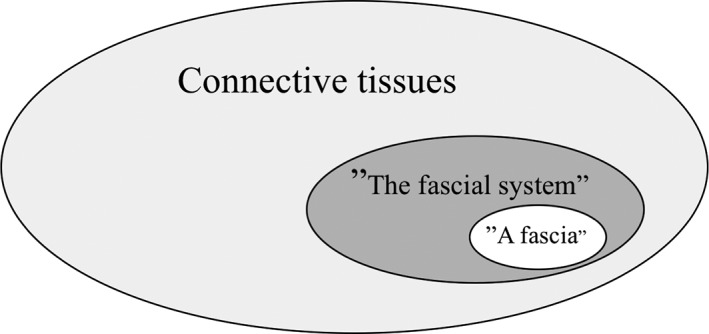
The nomenclature recommendations of the FNC are based on the understanding that the wider and more functional term “the fascial net” (which some authors replace by “fascial tissues”) describes a subset of tissues belonging to the connective tissue system of the body. Similarly, the term “a fascia” (also called “proper fascia” by some authors) describes a subset of tissues within the larger category of “the fascial system.”

The first term “a fascia,” therefore, describes a subset of dense planar tissues within the larger tissue group described as “the fascial system,” which again can be understood as a subset within the even larger group of tissues that are described as “connective tissues” in medicine (Fig. 2).

The FNC considers this process to be an ongoing task. New anatomical research findings or novel decisions by other appointed medical nomenclature groups, such as the Federative Committee on Anatomical Terminology, will conceivably constitute sufficient reason for conducting subsequent Delphi process rounds among the available experts and for discussing possible future amendments. As always have been, contributions from additional experts in the field will be welcome at any point of time during this process.

## References

[ca23423-bib-0001] Adler M, Ziglio E, (eds.). 1996 Gazing into the Oracle: The Delphi Method and its Application to Social Policy and Public Health. London: Kingsley Publishers.

[ca23423-bib-0003] Adstrum S , Hedley G , Schleip R , Stecco C , Yucesoy C . 2017 Defining the fascial system. J Bodyw Mov Ther 21:173–177. 28167173 10.1016/j.jbmt.2016.11.003

[ca23423-bib-0004] Benetazzo L , Bizzego A , De Caro R , Frigo G , Guidolin D , Stecco C . 2011 3D reconstruction of the crural and thoracolumbar fasciae. Surg Radiol Anat 33:855–862. 21203765 10.1007/s00276-010-0757-7

[ca23423-bib-0005] Benjamin M . 2009 The fascia of the limbs and back: A review. J Anat 214:1–18. 19166469 10.1111/j.1469-7580.2008.01011.x PMC2667913

[ca23423-bib-0006] Benjamin M , Kaiser E , Milz S . 2008 Structure‐function relationships in tendons: A review. J Anat 212:211–228. 18304204 10.1111/j.1469-7580.2008.00864.x PMC2408985

[ca23423-bib-0007] Chaitow L, Findley TW, Schleip R, (eds.). 2012 Fascia Research III: Basic Science and Implications for Conventional and Complementary Health Care. Munich: Kiener.

[ca23423-bib-0008] Federative Committee on Anatomical Terminology [FCAT] . 1998 Terminologia Anatomica: International Anatomical Terminology. New York: Thieme.

[ca23423-bib-0009] Findley TW . 2011 Fascia research from a clinician/Scientist's perspective. Int J Ther Massage Bodywork 4:1–6. 10.3822/ijtmb.v4i4.158 PMC3242643 22211151

[ca23423-bib-0010] Findley TW, Schleip R, (eds.). 2007 Fascia Research: Basic Science and Implications for Conventional and Complementary Health Care. Munich: Elsevier Urban & Fischer.

[ca23423-bib-0011] von der Gracht H . 2012 Consensus measurement in Delphi studies—Review and implications for future quality assurance. Technol Forecast Soc Change 79:1525–1536.

[ca23423-bib-0012] Guimberteau JC , Armstrong C . 2015 Architecture of Human Living Fascia: The Extracellular Matrix and Cells Revealed through Endoscopy. Edinburgh: Handspring Publishing.

[ca23423-bib-0013] Huijing PA . 1999 Muscle as a collagen fiber reinforced composite: A review of force transmission in muscle and whole limb. J Biomech 32:329–345. 10213024 10.1016/s0021-9290(98)00186-9

[ca23423-bib-0014] Huijing PO , Langevin HM . 2009 Communicating about fascia: History, pitfalls and recommendations. Int J Ther Massage Bodywork 2:3–8. 10.3822/ijtmb.v2i4.63 PMC3091474 21589739

[ca23423-bib-0015] Huijing P, Hollander P, Findley TW, Schleip R, (eds.). 2009 Fascia Research II: Basic Science and Implications for Conventional and Complementary Health Care. Munich: Elsevier Urban & Fischer.

[ca23423-bib-0016] Landers K , 2019 Fascia: The Mysterious Tissue. Blog post. URL: http://www.n2physicaltherapy.com/blog/fascia-mysterious-tissue/. [accessed April 15, 2019].

[ca23423-bib-0017] Langevin H . 2014 Langevin's response to Stecco's fascial nomenclature editorial. J Bodyw Mov Ther 18:444. 25042318 10.1016/j.jbmt.2014.04.016

[ca23423-bib-0033] Netter FH . 2011 Atlas of Human Anatomy. 5th Ed. Philadelphia: Saunders Elsevier.

[ca23423-bib-0018] Paoletti S . 2006 The Fasciae: Anatomy, Dysfunction & Treatment. Seattle, WA: Eastland Press.

[ca23423-bib-0032] Platzer W . 2008 Color Atlas of Human Anatomy. Vol. 1 6th Ed. New York: Thieme Inc.

[ca23423-bib-0019] Schleip R , Jäger H , Klingler W . 2012 What is ‘fascia’? A review of different terminologies. J Bodyw Mov Ther 16:496–502. 23036881 10.1016/j.jbmt.2012.08.001

[ca23423-bib-0020] Schwind P . 2006 Fascial and membrane technique: A manual for comprehensive treatment of the connective tissue system Urban & Fischer Verlag. Germany: Muenchen.

[ca23423-bib-0021] Standring S, (ed.). 2008 Gray's Anatomy: The Anatomical Basis of Clinical Practice. 39th Ed. Edinburgh: Elsevier.

[ca23423-bib-0022] Standring S, (ed.). 2015 Gray's Anatomy: The Anatomical Basis of Clinical Practice. 40th Ed. Edinburgh: Elsevier.

[ca23423-bib-0023] Stecco C . 2014 Why are there so many discussions about the nomenclature of fasciae? J Bodyw Mov Ther 18:441–442. 25042316 10.1016/j.jbmt.2014.04.013

[ca23423-bib-0024] Stecco C , Schleip R . 2016 A fascia and the fascial system. J Bodyw Mov Ther 20:139–140. 26891649 10.1016/j.jbmt.2015.11.012

[ca23423-bib-0025] Stecco C , Adstrum S , Hedley G , Schleip R , Yucesoy CA . 2018 Update on fascial nomenclature. J Bodyw Mov Ther 22:354. 29861233 10.1016/j.jbmt.2017.12.015

[ca23423-bib-0034] Tank PW . 2012 Grant's Dissector. 15th Ed. Philadelphia: Lippincott Williams & Wilkins.

[ca23423-bib-0026] van der Wal J . 2009 The architecture of the connective tissue in the musculoskeletal system—An often overlooked functional parameter as to proprioception in the locomotor apparatus. Int J Ther Massage Bodyw 2:9–23. 10.3822/ijtmb.v2i4.62 PMC3091473 21589740

[ca23423-bib-0027] Wearing SC, Schleip R, Chaitow L, Klinger W, Findley T, (eds.). 2015 Fascia Research IV: Basic Science and Implications for Conventional and Complementary Health Care. Munich: Kiener.

[ca23423-bib-0028] Wendell‐Smith P . 1997 Fascia: An illustrative problem in international terminology. Surg Radiologic Anat 19:273–277. 10.1007/BF01637586 9413070

[ca23423-bib-0029] Wilke J , Schleip R , Yucesoy CA , Winfried B . 2018 Not merely a protective packing organ? A review of fascia and its force transmission capacity. J Appl Physiol 124:234–244. 29122963 10.1152/japplphysiol.00565.2017

[ca23423-bib-0030] Willard FH , Vleeming A , Schuenke MD , Danneels L , Schleip R . 2012 The thoracolumbar fascia: Anatomy, function and clinical considerations. J Anat 221:507–536. 22630613 10.1111/j.1469-7580.2012.01511.x PMC3512278

[ca23423-bib-0031] Yucesoy CA , Huijing PA . 2007 Substantial effects of epimuscular myofascial force transmission on muscular mechanics have major implications on spastic muscle and remedial surgery. J Electromyogr Kinesiol 17:664–679. 17395489 10.1016/j.jelekin.2007.02.008

